# THE EFFICACY OF TOPICAL DIPHENCYPRONE IN THE TREATMENT OF ALOPECIA AREATA

**DOI:** 10.4103/0019-5154.49001

**Published:** 2009

**Authors:** Akhyani Maryam, Seirafi Hassan, Farnaghi Farshad, Banan Parastoo, Lajevardi Vahide

**Affiliations:** *From the Department of Dermatology, Razi Hospital, Tehran University of Medical, Sciences, Tehran, Iran. E-mail: vahide_Lajevardi@yahoo.com*

Sir,

In a retrospective study, 54 patients (38 females and 16 males) with chronic extensive alopecia areata, who had used diphencyprone (DPCP) for more than 1.5 years, between January of 2001 and December of 2005, were studied. The extent of hair loss was severe (>75%), moderate (26-75%), and mild (<25%) in 68.5%, 29.6%, and 1.9% of them, respectively. The mean duration of the disease was 7.8 ± 8.1 years.

The patients were sensitized initially with a 2% solution of DPCP in acetone, which was applied to a 5-centimeter-diameter circular area on the scalp. Treatment started two weeks after sensitization with 0.001% DPCP, on one-half of the patient's scalp. The concentration of the solution used was increased stepwise (0.01%, 0.1%, 0.2%, 0.5%, 1% and 2%) until redness, pruritus, but without vesicular eruption maintained. The patients were instructed to avoid direct exposure of the scalp to sun and not to wash the scalp for 48 hours after DPCP treatment. If terminal hair growth was noted, the entire scalp was treated under the same weekly protocol.

**Figure 1:**
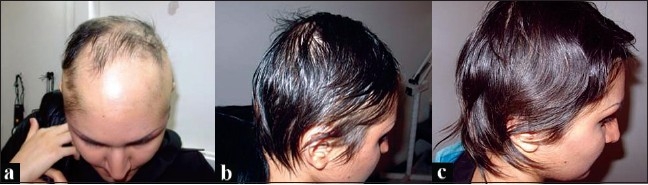
(a) 12 months after treatment; (b) 15 months after treatment; (c) 18 months after treatment

**Figure 2:**
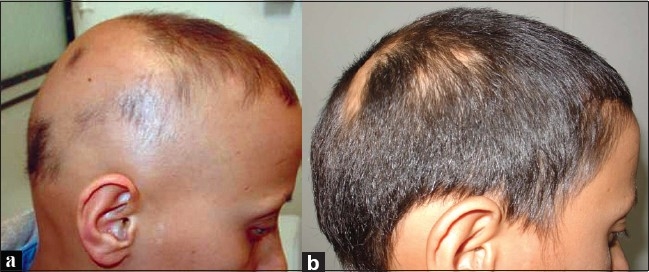
(a) Before treatment; (b) Six months after treatment

**Figure 3:**
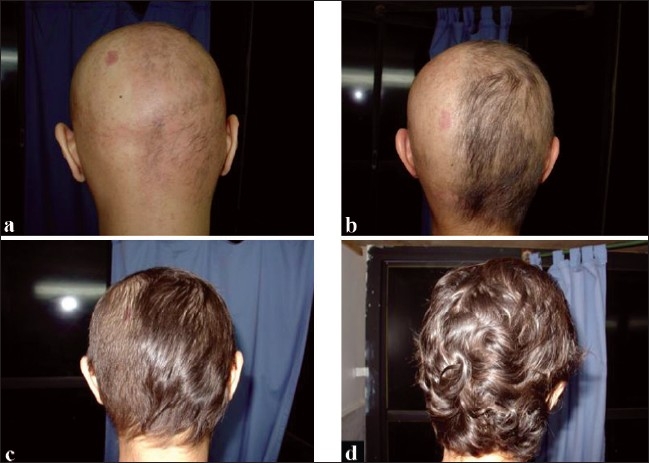
(a) 3 months after sensitization; (b) 3.5 months after sensitization; (c) 5 months after sensitization; (d) 7.5 month after sensitization

Terminal hair regrowth on the scalp was excellent (76-100%) in 40.7%, good (51-75%) in 14.8%, moderate (26-50%) in 14.8%, and mild (<25%) in 29.6% of patients [Figures 1–3].

Sixty two percent of the patients, with a duration of ≤10 years, had good and/or excellent response to the treatment (*P* = 0.017).

There was no relationship between response to treatment and sex, onset of disease, nail involvement, atopy, extent of hair loss, and family history of alopecia areata.

During the treatment, 33% of the patients experienced relapse. Continuation of the DPCP therapy caused hair re-growth in 21 of them. Adverse effects included contact dermatitis on the face or neck (five of 54), hyperpigmentation (four of 54) and occipital lymphadenopathy (one of 54).

In this study, the response to the treatment in 55.5% of the patients was appropriate. In other reports, however, the percentage of success varied greatly from four to 85%.[1] The changes in response rates may be due to the number of patients in trials, the type, duration and severity of the alopecia areata, and different methods of assessing clinical efficacy.

In addition, in our study, duration of disease was the only predictor for response rate. The presence of nail changes, a personal history of atopy, long duration of alopecia before treatment, baseline extent of alopecia, age at disease onset and duration of treatment have been considered as prognostic factors in other studies.[2,4]

It seems that the topical DPCP in the treatment of severe alopecia areata is effective, but with a slightly high relapse rate during treatment. Duration of the disease, less than 10 years, is a main predictor for this response rate.
